# Thoracic pedicle subtraction osteotomy for correction of proximal junctional kyphosis after surgery for adolescent idiopathic scoliosis: A case report

**DOI:** 10.1016/j.ijscr.2020.01.025

**Published:** 2020-01-27

**Authors:** Jose Ramírez-Villaescusa, Isabel Cambronero Honrubia, David Ruiz-Picazo, Jesús López-Torres Hidalgo, Ernesto González Rodriguez

**Affiliations:** a Department of Orthopedics and Traumatology, Spine Unit, Albacete University Teaching Hospital, Albacete, Spain; b Castilla-La Mancha Health Service (Servicio de Salud de Castilla-La Mancha), University of Castilla-La Mancha, Spain; c Department of Orthopedics and Traumatology, Spine Unit, Morales-Messeguer Teaching Hospital, Murcia, Spain

**Keywords:** Adolescent idiopathic scoliosis, Thoracic pedicle subtraction osteotomy, Proximal thoracic kyphosis deformity, Spinal surgery, Case report

## Abstract

**Introduction:**

Complications in the upper thoracic spine are not uncommon after corrective surgery for deformities in adults and adolescents. Proximal junctional failure has been linked to structural osseous or ligamentous failure and proximal junctional kyphosis has been described as an increase in preoperative proximal kyphosis.

**Case description:**

A 20-year-old male patient intervened after atypical development of idiopathic scoliosis, with rapid progression nearing skeletal maturity. While an increase in the magnitude of the main thoracic curve in the coronal plane was observed, the progression of structural sagittal plane deformity of the proximal thoracic curve was not identified due to poor visualization. This resulted in improper identification of curve type and choice of fusion levels, with progressive residual kyphosis across follow-up. At the age of 27, the patient was re-intervened by means of pedicle subtraction osteotomy in the apical area of the proximal thoracic deformity. Although an adequate correction was achieved, the remaining deformity of 50° and the proximal failure required extending the instrumentation and fusion to the cervical spine. This has shown itself to be an effective technique for correction of proximal residual or progressive symptomatic fixed kyphosis, thereby avoiding the morbidity of the anterior or combined approaches.

**Conclusions:**

In adolescent deformity, an adequate preoperative planning including clinical and radiological study must be carried out, paying special attention to the sagittal plane to identify major and minor structural curves.

The pedicle subtraction osteotomy, despite being a demanding technique and not entirely risk-free, has shown itself to be an effective corrective technique.

## Highlights

• Proximal junctional kyphosis in adolescent idiopathic scoliosis is frequent.
• Poor visualization of the sagittal plane leads to incorrect identification of curve type.
• Residual or progressive symptomatic kyphosis may require surgical treatment.
• Pedicle subtraction osteotomy may be an effective corrective technique.

## 1 Introduction

Complications in the proximal area among patients who undergo corrective surgery for adult and adolescent deformities are frequent. Proximal junctional failure (PJF) has been described as an increase in kyphosis associated with structural injury. Proximal junctional kyphosis (PJK) is defined as an increase in proximal kyphosis of 10° or more between the upper-most instrumented vertebra and the two supra-adjacent levels. Prevalence of PJK has been described with wide range of variability in adults (20–39%) and in adolescents (0–55%), following surgery deformity [[1], [2], [3]].

In adolescent scoliosis, both major and minor structural curves in the coronal and sagittal plane should be included in the fusion [4]. In the analysis of the proximal thoracic curve, poor visualisation of the sagittal plane is not uncommon [5], with incorrect identification of curve type and thus improper choice of fusion levels, which can then influence the persistence or progression of residual kyphosis.

Treatment options range from observation in cases of kyphosis without clinical repercussions to the need for surgery in cases involving pain, progressive deformity or neurologic injury. Pedicle subtraction osteotomy (PSO) by single posterior approach has shown great corrective capacity in cases of severe sagittal deformity, thereby avoiding the morbidity of the anterior or combined approach [[6], [7], [8]].

This paper reports an adolescent case of scoliosis with proximal structural kyphosis treated by means of PSO.

This work has been reported in line with the SCARE criteria [9].

## 2 Presentation of case

A 16-year-old male patient was referred to the traumatology outpatient clinic for spinal deformity assessment. The patient, who had been previously diagnosed with moderate mental retardation due to perinatal anoxia, was co-operative during the examination, with no gait alterations.

The physical examination showed deformity of the thoracic spine with gibbous deformity, which was accentuated with Adam’s forward bend test. The patient presented no asymmetry of shoulders, thoracolumbar triangles or dissymmetry of lower limbs. The neurologic examination indicated no neurologic deficit in the lower extremities or pathologic reflexes.

**Fig. 1:**
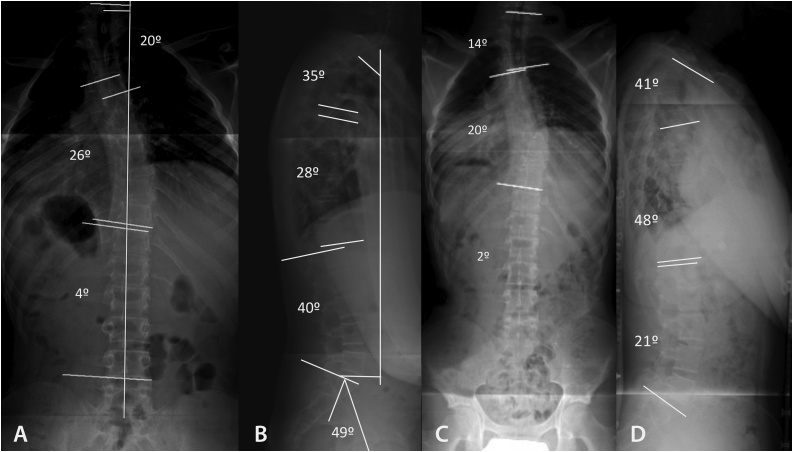
Radiologic study of the full spine in upright standing anteroposterior and lateral view. A, B: At ages 18 and 19 years, proximal thoracic (PT), main thoracic (MT), and thoracolumbar/lumbar (TL/L) curves were low magnitude non-structural curves and they were treated by observation and brace (C, D).

**Fig. 2:**
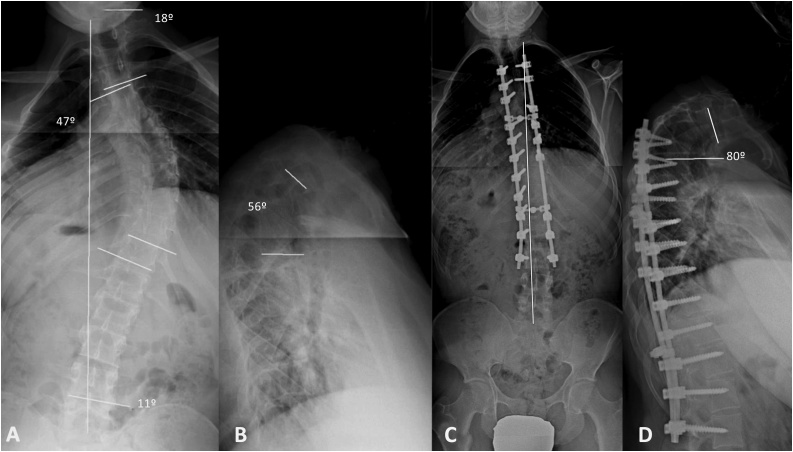
Radiologic study in anteroposterior and lateral view. A: At the age of 20 years, progression of the deformity can be observed with increase in the magnitude of the main thoracic (MT) curve (47°). B: In the lateral plane, the proximal thoracic (PT) curve of 56° (T2–T5) was not included in the fusion with T4 being the level of proximal fusion. C, D: Postoperative image in the follow-up at 7 years with proximal kyphosis of 80°.

The radiologic study of the full spine in upright standing anteroposterior view showed the triradiate cartilage closed and the degree of iliac ossification was Risser 3. We observed a proximal thoracic (PT) curve of 20°, a main thoracic (MT) curve of 26° and a thoracolumbar curve (TL/L) of 4°, as measured by the Cobb method and coronal imbalance 1,5 cm (vertical line center sacrum) (Fig. 1A). The lateral view showed proximal thoracic kyphosis at T2–T5 of 35°, thoracic kyphosis at T5–T12 of 28°, lumbar lordosis at L1-S1 of 21° and adequate sagittal balance (sagittal vertebral axis plumb line C7-S1 1.5 cm (Fig. 1B). At age 19 years old with Risser 3–4, the curves were observed to maintain a similar magnitude in the coronal plane (PT of 14°, MT of 20° and TL/L of 2°) (Fig. 1C), with an increase in the magnitude of curves in the sagittal plane (T2–T5 of 41°, T5–T12 of 48° and LL of 21°) (Fig. 1D). At age 20 years old with Risser 4–5, there was evidence of rapid progression of the clinical deformity (PT 18°), (MT 47°) (Fig. 2A) and upper sagittal thoracic deformity T2–T5 of 56° (Fig. 2B). It was decided that the increase in the clinical deformity and radiologic magnitude of the curve called for surgical treatment. The intervention was performed under general anesthesia, neurophysiologic monitoring of somatosensory evoked potentials (SSEPs) and motor evoked potentials (MEPs), by means of the posterior approach, using free-hand technique at levels T4-L3. No screws were placed at T7 left concave and T5, T10, T11 and T12 in convex right sides. The correction was performed using conventional maneuvers and T4-L3 posterior fusion with corticocancellous autograft obtained from the surgical field.

**Fig. 3:**
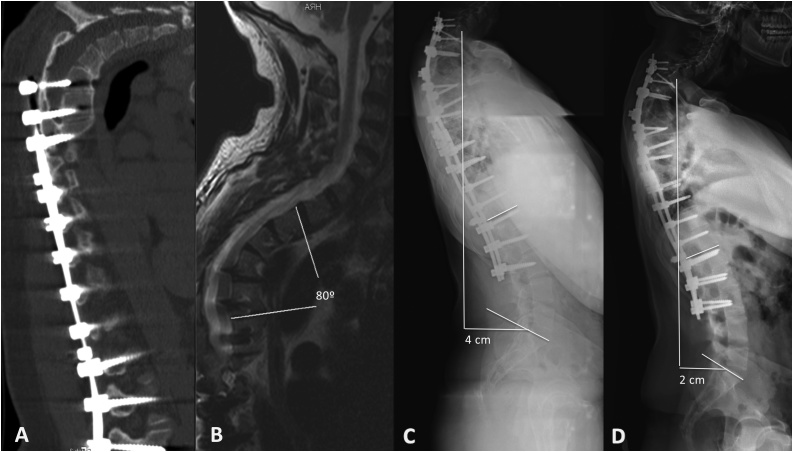
A: Sagittal images of the computed tomography (CT) study, with proximal kyphosis of 80° T2–T5 without correction in decubitus position. B: Sagittal images of the magnetic resonance (MR) study, in which no spinal changes are observed in the apical thoracic region. C: Postoperative radiologic study after performance of pedicle subtraction osteotomy at the level of the apical region of the deformity (T3), with proximal extension of the instrumentation showing adequate correction of 30° with residual deformity of 50°. Negative sagittal balance (sagittal vertebral axis) (SVA) of 4 cm. D: Twelve months after the intervention the patient referred pain and cervicothoracic area showed deformity and swelling. In radiological study, pulling out at C7 level and T1–T4 kyphosis of 53° were observed, but findings suggested fused PSO level. Radiologic study showed pulling out at C7 level and low increase of T1–T4 kyphosis (53°) and negative sagittal balance (sagittal vertebral axis) (SVA) of 2 cm.

Postoperative radiography showed correction of the main thoracic curve of 90% but residual deformity in the lateral plane with proximal kyphosis at T1–T4 of 70°, which was well tolerated. After 7 years of follow-up, progression of the proximal kyphosis (80°) was observed in the radiological study (Fig. 2D). The computed tomography (CT) (Fig. 3A) and magnetic resonance (MR) (Fig. 3B) showed rigidity of the curve without correction in decubitus position, absence of proximal structural failure due to fracture of the upper instrumented vertebra or ligamentous injury and no compression signs or abnormalities in the spinal cord.

A new intervention was performed at age 27 years old using neurophysiologic monitoring of SSEPs and MEPs and cranial traction by placement of a Mayfield head holder. A PSO at T3 level was performed to extend the proximal instrumentation using lateral mass screws at C7 and pedicle screws at T1 and T2 levels. Any spinal cord changes in SSEPs and MEPs were observed during the reduction maneuvers.

The radiologic study showed a correction of 30° with a residual kyphosis of 50°. Proper coronal balance was achieved but negative sagittal balance (SVA 4 cm) was observed at eleven months of the intervention (Fig. 3C).

However, twelve months after the intervention, the patient referred pain and cervicothoracic area showed deformity and swelling. In radiological study pulling out at C7 level screws, T1–T4 kyphosis of 53° and negative sagittal balance (SVA 2 cm) were observed (Fig. 3D), but findings suggested fused PSO level. To prevent deformity progression and complications, we extended the upper instrumentation to cervical spine. By angiography-CT, anomalous route of vertebral artery was ruled out and screws were placed at C2 pars interarticularis, lateral masses of C3–C6 lateral masses and C7 pedicle. A Ponte osteotomy at T1–T2 was performed to reduce kyphosis. Any intraoperative neurological events were detected.

**Fig. 4:**
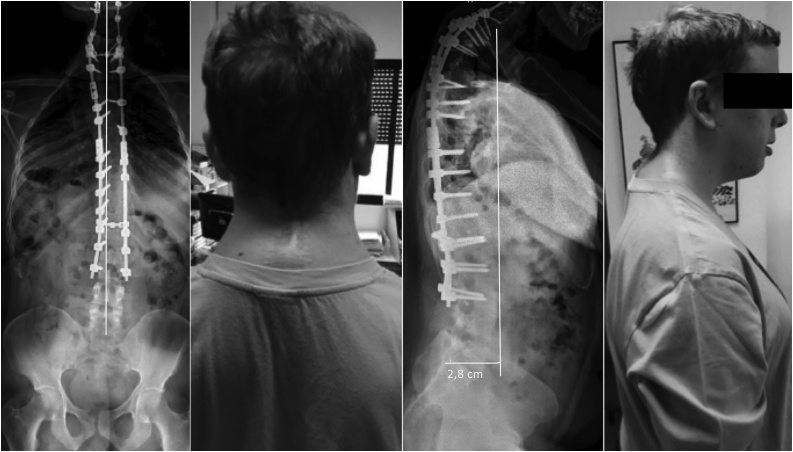
To prevent progression of deformity and complications we extended the upper instrumentation to cervical spine. By CT angiography, anomalous route of vertebral artery was ruled out and screws were placed at C2 pars interarticularis, lateral masses of C3–C6 lateral masses and C7 pedicle. A Ponte osteotomy at T1–T2 was performed to reduce kyphosis.

At the one-year follow-up visit, the patient did not report pain or functional loss and a radiological study showed a well-tolerated an upper 53° kyphosis at T1–T4 and coronal and sagittal balance (Fig. 4).

## 3 Discussion

In patients with adolescent idiopathic scoliosis (AIS), deformity progression has been related to the magnitude of the curve at the stage when pubertal growth and skeletal maturity begin to peak. Similarly, when approaching skeletal maturity, the risk of progression is negligible, though progression has been described in some Risser-4 cases, most frequently in men. AIS curves progress during the rapid growth period of the patient. While most curves decrease their progression significantly at the time of skeletal maturity, some continue to progress during adulthood, especially curves greater than 60° [10]. In this case, an atypical evolution with rapid progression was observed in the magnitude of the main thoracic curve in the coronal plane and upper thoracic kyphosis in the sagittal plane, when the patient was approaching skeletal maturity. The case is described as idiopathic scoliosis, since the patient had no gait disturbances, trunk deformity or pelvic inclination. However, the history of perinatal anoxia, the rapid increase of progression of coronal and proximal kyphosis deformities at the end of growth could be related to neuromuscular scoliosis [11].

A deformity pattern must be identified in both coronal and sagittal planes in order to select the fusion levels. To prevent mistakes, if upper thoracic sagittal structural curve is suspected, a supine hyperextension crosstable lateral x-ray (bolster under apical kyphosis) should be performed [5,12]. If available, the EOS device provides image acquisition and reconstruction software provides accurate 3D spinal representations of scoliotic spinal deformities with low-dose x-ray as this has the capacity to allow 3D spine reconstructions to be created from biplanar standing radiographs. [13]. In our case, an incorrect curve pattern was identified (Lenke Type 1) when should be considered Lenke Type 2 due to a structural upper sagittal thoracic kyphosis. The risk factors of PJK are related with posterior-approach surgery, the use of posterior instrumentation and preoperative thoracic hyperkyphosis [[14], [15], [16]]. Moreover, as in our case, other factors can be found to increase deformity. These can be the following: inadequate upper selection level, the structural upper sagittal deformity, great correction of coronal deformity, low instrumented vertebra below L2, no maintenance of previous physiological kyphosis (contouring rod in concave and convex side was lost) and negative compensatory postoperative sagittal imbalance. Inadequate restoration of thoracic kyphosis can result in PJK upper thoracic or cervical spine like flatback syndrome in adults with lumbar hypolordosis [1,17].

Corrective techniques with osteotomies by single posterior approach have yielded satisfactory results, avoiding the morbidity of the anterior and/or combined approach. The choice of the osteotomy type depends on the site, flexibility of the curve and magnitude of the correction [8]. While Ponte osteotomy ideally corrects 10° per treated level, and VCR is performed in severe PJK, the pedicle subtraction osteotomy (PSO) is used to restore up to 30°–35° per treated level. PSO has proven effective for correction of fixed sagittal deformity, although complications are frequent and potentially severe both perioperative (e.g., excessive bleeding and neurologic injury) and those later on (e.g., mechanical failures with breakage of implants), with a high number of reinterventions [8,18]. In this case, the PSO was performed at the apex of the deformity at T3 vertebra. Although proximal extension of a greater number of segments may be required in adult patients, our patient’s good bone quality made it possible to extend the instrumentation only three proximal levels, with lateral mass screws and pedicle screws at C7 and T1 and T2 levels respectively. However, although the patient was young and adequate correction was achieved decreasing 30° the preoperative kyphosis, previous tendency to chin on chest and a 50° of residual deformity in some patients, can be risk factors to proximal failure. Only three upper levels to PSO could not be enough to prevent failure and long upper instrumentation should be taken into account.

Our case study has shown a way for avoiding errors by using proper clinical evaluation and radiologic assessment to determine fusion levels to prevent any residual deformity that might require a new intervention.

## Funding

This research did not receive specific grant form funding agencies in the public, commercial, or not-for-profit sectors.

## Ethical approval

The paper is a case report, and therefore does not require ethics approval.

## Consent

Informed consent has been obtained from the patient, and all identifying details have been omitted.

## Author contribution

Isabel Cambronero – Honrubia Acquisition of data, management of case.

José Ramírez-Villaescusa – Conception of study, acquisition and analysis of data.

David Ruiz-Picazo – Revision and drafting the article.

Jesús López-Torres Hidalgo – Revision of article.

Ernesto González-Rodriguez – Revision of article.

## Registration of research studies

This case report is not a human study so is not necessary a registration of research studies.

## Guarantor

The guarantor of this article is Dr. Ramírez-Villaescusa.

## Provenance and peer review

Not commissioned, externally peer-reviewed.

## Declaration of Competing Interest

We wish to confirm that there are no conflicts of interest associated with this publication.
